# Supplementary material for: Splice-Junction-Based Mapping of Alternative Isoforms in the Human Proteome
Source: Cell Rep. Author manuscript; Available in PMC 2020 Jan 15. (PMC6961840; doi:10.1016/j.celrep.2019.11.026)
Supplement: 2 [file NIHMS1546469-supplement-2.zip › DF1/PXD010154/Heart/Heart_84_ASB8_EYLLLLSR.pdf]

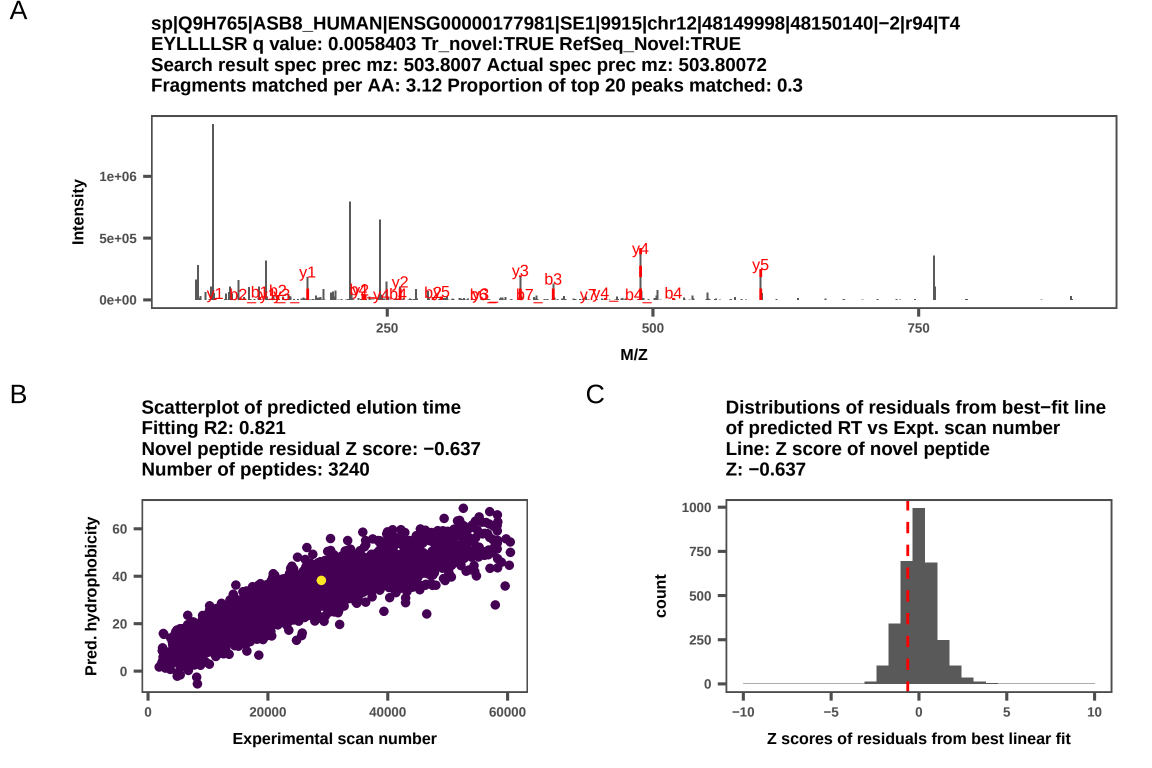
A
sp|Q9H765|ASB8_HUMAN|ENSG00000177981|SE1|9915|chr12|48149998|48150140|−2|r94|T4
EYLLLLSR q value: 0.0058403 Tr_novel:TRUE RefSeq_Novel:TRUE
Search result spec prec mz: 503.8007 Actual spec prec mz: 503.80072
Fragments matched per AA: 3.12 Proportion of top 20 peaks matched: 0.3
Intensity
1e+06
5e+05
0e+00
y1
y2
y3
y4
y5
b3
b4
250
500
750
M/Z
B
Scatterplot of predicted elution time
Fitting R2: 0.821
Novel peptide residual Z score: −0.637
Number of peptides: 3240
Pred. hydrophobicity
60
40
20
0
0
20000
40000
60000
Experimental scan number
C
Distributions of residuals from best−fit line
of predicted RT vs Expt. scan number
Line: Z score of novel peptide
Z: −0.637
count
1000
750
500
250
0
−10
−5
0
5
10
Z scores of residuals from best linear fit
